# Modeling the Demand for Shared E-Scooter Services

**DOI:** 10.1177/03611981211051620

**Published:** 2021-10-21

**Authors:** Muntahith Mehadil Orvin, Jashan Kaur Bachhal, Mahmudur Rahman Fatmi

**Affiliations:** 1 School of Engineering, Department of Civil Engineering, University of British Columbia, Kelowna, BC, Canada; 2 School of Engineering, Department of Mechanical Engineering, University of British Columbia, Kelowna, BC, Canada

**Keywords:** planning and analysis, effects of information and communication technologies (ICT) on travel choices, emerging, shared mobility, transportation demand forecasting, demand estimation, models/modeling

## Abstract

This paper presents the findings on modeling the demand for shared e-scooter services (SES); specifically, spatio-temporal variation of SES demand. A zero-inflated negative binomial (ZINB) model is developed using the count data of trip origins at the dissemination area level from Kelowna, Canada. The motivation for adopting the ZINB model is the presence of excess zeros in the count data. ZINB has two components: the zero-inflated component accounts for excess zeros, and the count component accounts for the over-dispersion characteristics of data resulting from excess zeros. In addition to the ZINB, several other count models including hurdle models are estimated. The goodness-of-fit measures suggest that the ZINB model outperforms other methods. The model results confirm the effects of temporal, weather, transportation infrastructure, land use, and neighborhood characteristics. For example, the count model results reveal that SES demand is more likely to be higher during summer, mid-day on weekends, afternoons of weekdays, and days without rainfall. Furthermore, higher e-scooter index, higher density of cycle tracks, heterogeneous land use, urban centers, lower elevation, and neighborhoods with higher density of hotels and younger population might induce higher demand. The zero component results of the model are consistent with the findings revealed by the count component. The model is validated using a hold-out sample, and the validation results confirm that the prediction performance of the model is reasonably satisfactory. The findings of this study provide important insights into when and where the demand is higher, which will assist in effective policy-making supporting e-scooter use.

Micro-mobility has emerged as a viable and sustainable alternative to automobiles for traveling shorter distances (*1*). Shared micro-mobility services such as bikes, scooters, skateboards, e-bikes, and e-scooters are emerging as they enable individuals to have short-term access to a travel option on an as-needed basis (*2*). These shared services have gained huge popularity recently for their convenience, sustainability, affordability, and efficiency. One of the latest additions to the shared micro-mobility system is the shared e-scooter service (SES). SES is a comparatively fast and convenient mode for traveling shorter distances. It is usually dockless, implying that scooters can be picked up or dropped off at any location in the permitted service areas. A recent standing version of SES was initially launched in 2017. Bird was the first company that started operating SES in North America (*3*). Since then SES has expanded immensely because it is not only convenient but also a key solution to the first/last mile mobility issue in urban areas (*4*, *5*). SES has also emerged as a popular micro-mobility mode to perform recreational activities (*6*). As a result, cities around the world are investing in promoting newly emerging shared micro-mobility services such as SES. Companies such as Scoot, OGO, Zip, Roll, Bolt, and Bounce have launched their e-scooters in various parts of the world to meet the growing demand for SES. However, Severengiz et al. (*7*) argued that e-scooters might not be as sustainable as expected because of their potential to increase the life-cycle emissions compared with other modes of transport. They suggested that demand management initiatives such as limiting the usage of SES to within the inner-city area might be more environmentally sustainable because of reducing the distances traveled for the collection of e-scooters for charging. Therefore, to ensure the sustainability of the SES system, and develop effective plans and policies to promote SES, it is critical to understand the demand for this micro-mobility service. In particular, the research questions are: when is the demand? (i.e., the temporal variation of demand), and where is the demand? (i.e., the spatial variation of demand). Because of the novelty of SES, however, limited research has focused on the spatio-temporal variation of SES demand.

This study investigates the demand for SES by utilizing e-scooter count data from Kelowna, Canada. One of the key features of this study is to examine the combined spatio-temporal variation of SES demand. In the case of spatial characteristics, built environment attributes such as transportation infrastructure, land use, and neighborhood features are extensively tested. Temporal characteristics include the time of day, week, season, and weather attributes. A zero-inflated negative binomial (ZINB) model is developed using the zonal count of trips originating at the dissemination area level (DA). DA refers to the smallest unit of geographic area in Canada for which all the census data are disseminated. The motivation for adopting the ZINB model is the presence of excess zeros in the count data. The excess zeros are classified as structural (i.e., DAs always with zero counts) and sampling zeros (i.e., DAs with zero counts only at specific time periods). The over-dispersion attribute of data is also tackled within the ZINB framework.

## Literature Review

SES is a fairly recent addition to the shared micro-mobility services. It provides several advantages including reducing vehicular emissions and congestion, serving as first- and last-mile mobility option, and station-less service (i.e., flexibility to pick up and return e-scooters), among others (*6*, *8* [Bibr bibr9-03611981211051620]–*10*). Since Bird launched its e-scooter service in 2017, it has experienced rapid growth. For example, Bird recorded approximately 10 million rides in its maiden year of operation (*11*). However, literature on SES is limited. Among the few publications, the majority of the research related to SES has focused on its overall environmental impact, safety, and usage pattern in urban areas. For instance, Badeau et al. (*12*) examined the SES-related injuries in Salt Lake City, Utah. Their analysis revealed a remarkable increase in SES-related head and body muscle injuries between 2017 and 2018. Hardt and Bogenberger (*6*) examined the usage, trip purpose, and trip distance of the e-scooter users in Munich, Germany. Results revealed that SES is mostly used for commuting and recreational activities. Mathew et al. (*4*) explored the temporal usage of SES in Indianapolis. They found that SES is not generally used in the morning for commuting purposes. Results further suggested that about 85% of the e-scooters are used for less than an hour per day, revealing the usage of SES for shorter distance trips. To further promote SES as an alternative travel mode, effective planning and infrastructure investment decision-making are required. For effective plans and policies, it is critical to understand the demand for SES, which has not occurred to any significant extent.

In the domain of shared micro-mobility, several studies have attempted to analyze the demand. The majority of them have focused on bikeshare services, particularly focusing on the spatial factors (*13*, *14*). For example, Ma et al. (*14*) investigated the impact of bike-sharing fleets, socio-demographic factors, and land use attributes on demand for dockless bikeshare (DBS) in Nanjing, China. One of the key findings is that the density of recreational activity points is positively associated with the demand for DBS. Several studies have focused on the combined influence of spatio-temporal factors on demand (*15* [Bibr bibr16-03611981211051620]–*17*). For instance, Shen et al. (*17*) analyzed the association of DBS demand with built environment, transit accessibility, bike infrastructure, time of day, and weather attributes in Singapore. Dense heterogeneous areas, road intersections, weekends, and late afternoons are found to have a positive relationship with the number of DBS.

Few studies, however, have investigated the demand for SES. One of the primary reasons is the new and emerging nature of this technology. Among the few studies, Jiao and Bai (*18*) examined the influence of built environment on the demand for SES in Austin, Texas. They suggested that transit facilities, shorter distance to the city center, bike infrastructure, road connectivity, and population density are positively associated with SES trips. Bai and Jiao (*19*) extended this study and compared the results with Minneapolis. They found that the hotspot of SES usage was in the downtown areas in both cities. They further revealed that proximity to the city center, better access to transit, and heterogeneous land mix are more likely to have a positive influence on SES demand. Caspi et al. (*20*) explored the demand for SES in Austin. They suggested that the usage of SES is higher in areas with higher employment rates and bike infrastructures. Further research is required to investigate extensively the effects of spatial factors such as transportation infrastructure, land use, and neighborhood characteristics on SES demand. Incorporating the temporal effects is also required, since demand might vary temporally, such as over the day, week, season, and weather. Therefore, there is a gap in the literature on understanding the combined spatio-temporal variation of SES demand.

In the case of modeling methods, the majority of the previous studies analyzing demand for micro-mobility services have adopted regression modeling techniques using count data. For example, Shen et al. (*17*) developed autoregressive models and Ma et al. (*14*) utilized the ordinary least square and geographically weighted regression models to analyze the demand. In another study, spatial regression and geographically weighted regression models were developed (*20*). One of the challenges associated with the modeling of count data is the presence of over-dispersion characteristics. Several studies have adopted alternative modeling techniques such as Poisson (*21*) and negative binomial (NB) models to analyze the count data showing over-dispersion (*18*, *19*, *22*). However, a common reason for over-dispersion is the presence of excess zeros in the count data (*23*). This is a common challenge with zonal count data (*14*, *18*, *20*). To tackle the excess zero issue, previous studies either eliminated the counts with zero observations (*18*) or adopted a rule-based approach (*20*) to minimize the effects of excess zero counts. For example, Caspi et al. (*20*) used 200 m square grids and found 62% of grids having zero trips. They adopted a rule-based technique and considered only the count of grids that were within 3.5 km of the centroid of city center, since trip density was extremely high within the city center. However, the application of the model is restricted within the city center. Furthermore, ignoring excess zeros in the modeling framework might result in biased and inconsistent parameter estimates and poor prediction accuracy (*24*). This demands an extension of the conventional Poisson/NB model into advanced techniques such as zero-inflated (*24* [Bibr bibr25-03611981211051620]–*26*) and hurdle (*27*, *28*) models.

### Contributions of the Current Study

This study contributes by investigating the demand for SES. One of the key features of this study is to explore the spatio-temporal variation of SES demand. Temporal attributes such as time of day, week, season, and weather characteristics are tested. In the case of spatial characteristics, built environment attributes including transportation infrastructure, such as e-scooter index; land use, such as land use index; and neighborhood attributes, such as hotel density, are examined. Methodologically, a ZINB model is developed using the count data at the zonal level of DA in Kelowna, Canada. ZINB model is developed to tackle the over-dispersion characteristics of the count data resulting from excess zeros. The model is formulated as a two-component mixture model. The zero-inflated component is assumed to have a binary distribution. This component of the model accounts for excess zeros. The other component is a count model assuming a NB distribution.

## Data

### Sources of Data

The primary data source for this study is the shared mobility manager database collected from the City of Kelowna through the Populus platform. This portal includes information on all shared services in Kelowna, Canada. Populus uses two categories of data specifications: (i) General Bikeshare Feed Specification (GBFS) for live map, and (ii) Mobility Data Specification (MDS) for all other maps and historical data (*29*). This study utilizes the e-scooter database from this portal. This database provides the count of shared services trips originating and destined in Kelowna. This data also includes information about parking events of shared services. The counts of all deployed e-scooters are recorded from the MDS feed and aggregated over a specified time period at the zonal level. The smallest zonal level data available in Populus is DA. There are a total of 167 DAs in Kelowna. Temporarily, data is available at the following aggregate level: early morning (3:00–6:00 a.m.), morning (6:00–10:00 a.m.), mid-day (10:00 a.m.–3:00 p.m.), afternoon (3:00–7:00 p.m.), evening (7:00–10.00 p.m.), and night (10.00 p.m.–6:00 a.m.). Trips that are shorter than 10 seconds and longer than 10 hours are filtered out by Populus. This dataset does not include information on the availability of e-scooters in the DAs.

Secondary data are extracted from various resources. Transportation infrastructure and land use attributes are obtained from Kelowna Open Data. Transportation infrastructure-related data involves bike infrastructure, road networks, and bus stops, among others. Land use attributes include the percentage of areas classed as residential, commercial, park, aquatic, and urban center, among others. The location of activity points is collected from Desktop Mapping Technologies Inc. (DMTI). This information includes the location of food stores, educational services, health services, shopping centers, hotels, and recreational centers, among others. Neighborhood features are extracted from Statistics Canada. This data includes population density, dwelling density, dwelling type, average age, average household size, and employment rate, among others. Weather information is collected from Environment Canada. This data involves the historical temperature, wind speed, relative humidity, and rainfall, among others.

### Overview of SES Usage in Kelowna

**Figure 1.**
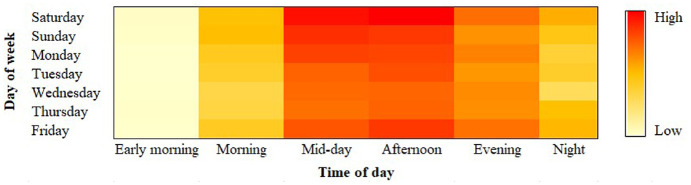
Percentage distribution of total shared e-scooter service (SES) demand in Kelowna by day of week and time of day.

SES started in Kelowna on July 13, 2019 and most of the operators were not active in the winter season. Therefore, this study utilizes data from July 13 to October 25, 2019. The majority of the SES companies (e.g., OGO, Bunny, and Zip) were fully functional during this time-frame. During this period, a total of 22,720 SES trips originated in Kelowna. The origin of the SES trips in any DA of Kelowna is dominated by the availability of e-scooters in that DA. E-scooters are removed from the streets at night, rebalanced, recharged, and returned within the city-authorized e-scooter network the next morning. The percentage share of total weekday and weekend trips per day was found to be 42% and 58%, respectively. Approximately 70% of trips were counted during the months of July and August, which is in the summer season in Kelowna. Figure 1 illustrates the percentage distribution of total SES demand over time of day and day of week in Kelowna. The analysis reveals that higher demand is observed during mid-day (i.e., around 34% of total trips) and afternoon (i.e., around 36% of total trips). Among these trips, a higher number of trips originated during the weekends. In contrast, the lowest demand was captured during the early morning period. Interestingly, demand was higher during the evening (i.e., 20% of total trips) compared with the morning (i.e., 4% of total trips) and night time (i.e., 5% of total trips).

**Figure 2.**
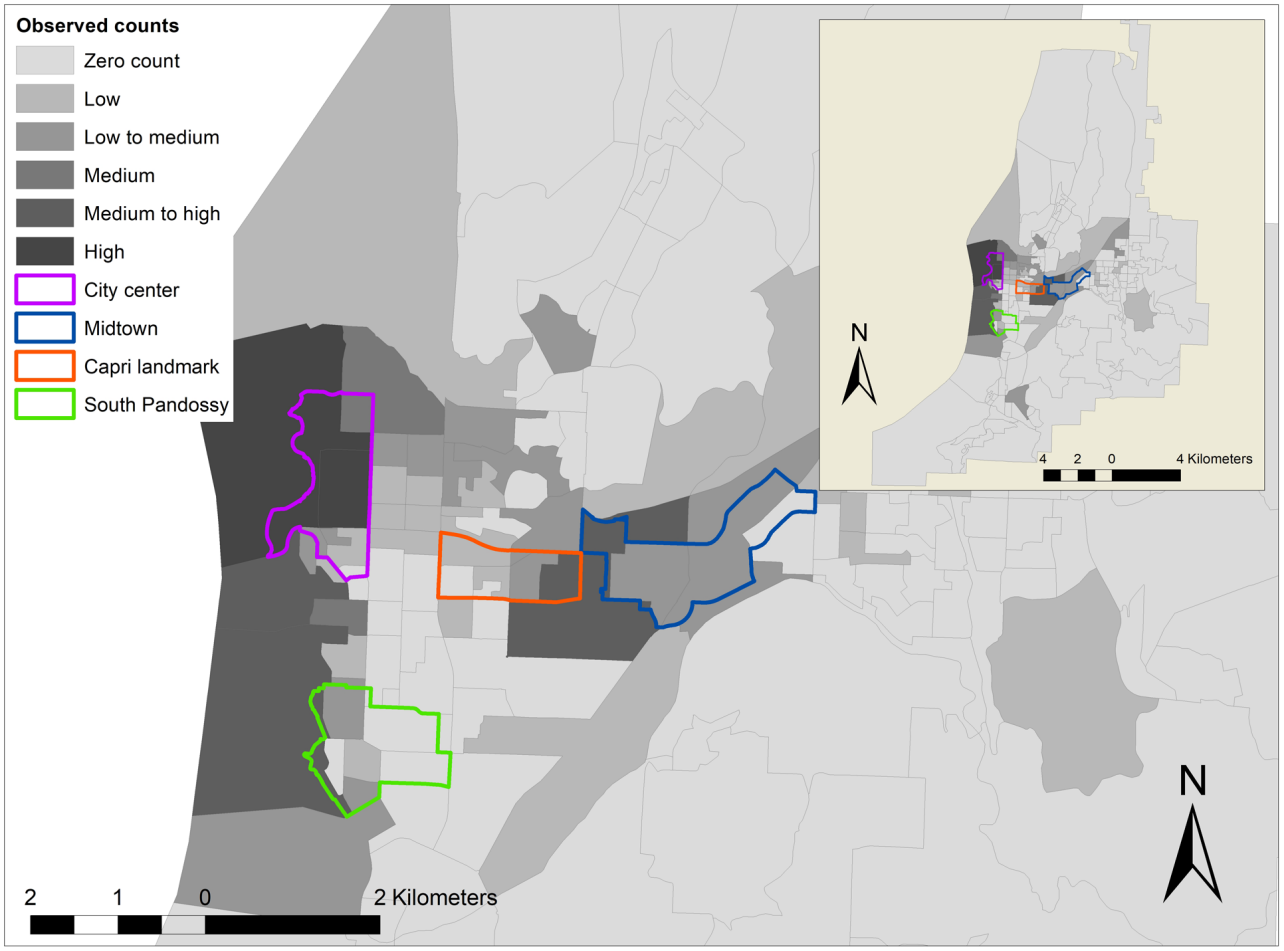
Observed shared e-scooter service (SES) demand in Kelowna.

The spatial unit of analysis for this study is the zonal level of the DA. Figure 2 shows the observed SES demand in Kelowna. It is evident that higher demand is revealed near the Okanagan Lake area adjacent to the downtown area of Kelowna. Furthermore, a secondary peak in demand is observed near the urban city center and the other urban centers such as Midtown, Capri landmark, and South Pandossy. The analysis suggests that around 91% of the total trips originate from DAs in the downtown and urban city center. Figure 2 further suggests that the majority of DAs have zero trip count. The excess zeros in the count data can be classified as either structural or sampling zeros. Structural zeros occur from DAs that always have zero counts. Out of the 167 DAs in Kelowna, 114 DAs are found to have zero count at all times. One of the reasons for structural zeros is the unavailability of e-scooters and bike infrastructure facilities in those DAs. On the other hand, sampling zeros might occur because of sampling variability. Sampling zero occurs because some DAs have zero counts only at specific periods.

### Explanatory Variables

This study extensively tests the effects of spatial and temporal attributes on SES demand. Spatial factors include transportation infrastructure, land use, and neighborhood attributes. All the explanatory variables are generated at the DA level using ArcGIS. Among the transportation infrastructure characteristics, an e-scooter index (EI) is generated using the collected transportation infrastructure attributes, following similar methodology to the bike index (BI) (*30*, *31*). This is because in Kelowna e-scooter rides are permitted only on bike infrastructures. The EI signifies the e-scooter friendliness of a location. EI is obtained by considering several indicators including road and bike infrastructure, topography, environment, and land use characteristics, among others. The EI value ranges from 0 to 1, where a value closer to 1 signifies higher e-scooter rideability and a value closer to 0 indicates lower rideability. Land use attributes are utilized to determine the land use index (LUI) (*32*). LUI refers to the diversity in land usage. LUI ranges on a scale of 0 to 1, where a value closer to 1 indicates diverse land uses. To examine the influence of road elevation, a slope raster is generated from the elevation data utilizing the ArcGIS spatial analyst tool. This provides an impression of the steepness of a DA by identifying the highest rate of change in the elevation value of the DA as compared with its neighboring DAs. The steeper the surface, the higher the percent of rise; which is calculated by the ratio of rise to run, expressed in percentages (*33*). Zonal statistics are used to detect the mean elevation for each DA from the percent rise raster. In addition, temporal and weather attributes are generated according to the time period of the observation.

## Methodology

This study develops a ZINB model to investigate the demand for SES using the trip counts originating at the zonal level of DAs in Kelowna. One of the purposes of developing a ZINB model is that it accounts for excess zeros in count data. However, in the case of count data analysis, Poisson regression models are predominantly used. The Poisson probability distribution can be expressed as:

(1) $$P(Z=Z_{i}|t_{i})=\frac{e^{-(t_{i}\mu_{i})}{\left(t_{i}\mu_{i}\right)}^{Z_{i}}}{Z_{i}!};Z_{i}=0,1,2\ldots n$$

where

$Z_{i}$ = number of SES trips originating in a DA for the *i*th observation,

$t_{i}$ = time interval, and

$\mu_{i}$ = mean number of SES count.

One of the limitations of the Poisson model is the restrictive assumption that conditional variance equals conditional mean. The count data of this study has a variance that is greater than the mean (i.e., over-dispersed). This limitation is relaxed in the NB modeling method (*24*). The NB model allows for over-dispersion by adding an error term to the mean of Poisson model as:

(2) $$\mu_{i}=e^{\left(\beta X_{i}+\varepsilon_{i}\right)}$$

where

β = vector of coefficient of the parameters (to be estimated),

*X* = set of explanatory variables, and

ε = error term

It is assumed that *exp*( $\varepsilon_{i})$ has a Gamma distribution with mean of 1 and variance *θ*. Thus, the conditional mean $\mu_{i}$ is different from the conditional variance of the count distribution $\mu_{i}(1+\theta \mu_{i})$ . The probability expression for the NB model is as follows:

(3) $$P(Z=Z_{i})=\frac{\Delta (Z_{i}+\theta^{-1})}{\Delta \left(Z_{i}+1\right)\Delta (\theta^{-1})}(\frac{1}{1+\theta \mu_{i}}{)}^{\frac{1}{\theta }}(1-\frac{1}{1+\theta \mu_{i}}{)}^{Z_{i}}$$

where

Δ(.) = Gamma function and

θ= NB dispersion parameter

One of the key reasons for over-dispersion is the existence of excess zeros. Excess zeros can be present because of structural and sampling zeros. Structural zeros are the zero counts that will always be zero, whereas sampling zeros refer to the zero counts that can be greater than zero, but appear to be zero because of sampling variability. The NB model is often limited to address the over-dispersion resulting from excess number of zero counts (*23*). Consequently, parameter estimation results might be biased and forecasting might be inconsistent for count data having excess zeros fitted with Poisson or NB models (*24*). Hurdle and zero-inflated models are generally developed to tackle the excess zeros. A hurdle model is usually utilized in the case of excess sampling zeros only. On the other hand, a zero-inflated NB model is used when data contains both excess structural and sampling zeros. Therefore, a zero-inflated model assumes that zero observations originate from both structural and sampling zeros (*25*).

The count data of this study include excess sampling and structural zeros. For example, sampling zeros occur when SES counts are not observed in a DA, however, counts are recorded for other time intervals. In contrast, DAs that never recorded SES counts are considered as structural zeros. Therefore, this study utilizes the ZINB modeling technique to analyze the SES count data. The ZINB model adopts a two-component mixture modeling approach. The first component is referred to as the zero-inflated component. Let a binary distribution with probability $\sigma_{i}$ that indicates whether SES counts are zero or not, then the overall probability of zero counts is the combined probability of structural and sampling zero outcomes. The sampling zeros are formed by the generic NB distribution which considers zero counts that occurred coincidentally. The final component is a typical NB count model with probability $1-\sigma_{i}$ . The probability distribution of the ZINB model can be expressed as:

(4) $$P\left(Z_{i}=k\right)=\left\{ \begin{array}{c} \sigma_{i}+\left(1-\sigma_{i}\right)f(Z_{i}=0),z=0\\ \left(1-\sigma_{i}\right)f(Z_{i}),z>0 \end{array}\right.$$

$f(Z_{i})$ is the NB distribution which takes the following form:

(5) $$f\left(Z_{i}\right)=P\left(Z=Z_{i}|\mu_{i},\theta \right)=\frac{\Delta (Z_{i}+\theta^{-1})}{\Delta \left(Z_{i}+1\right)\Delta (\theta^{-1})}(\frac{1}{1+\theta \mu_{i}}{)}^{\frac{1}{\theta }}(1-\frac{1}{1+\theta \mu_{i}}{)}^{Z_{i}};\mu_{i}=e^{\left(\beta X_{i}+\varepsilon_{i}\right)}$$

The ZINB model considers $\sigma_{i}$ as the logistic function which can be defined as:

(6) $$\mathrm{logit}\left(\sigma_{i}\right)=\omega Y_{i}$$

where ω = vector of coefficients for explanatory variables $Y_{i}$ in the zero-inflated component (to be estimated).

The parameters are estimated by maximizing the likelihood function. The log-likelihood function can be written as:

(7) $$\begin{array}{c} \mathrm{LL}=\sum_{i=1}^{N}\log\left(1+e^{\omega Y_{i}}\right)-\sum_{i:z_{i}=0}\log(e^{\omega Y_{i}}+(\frac{e^{\beta X_{i}}+\theta^{-1}}{\theta^{-1}}{)}^{-\theta^{-1}})\\ +\sum_{i:z_{i}>0}\left(\theta^{-1}\log\left(\frac{e^{\beta X_{i}}+\theta^{-1}}{\theta^{-1}}\right)+z_{i}\log\left(1+\theta^{-1}e^{-\beta X_{i}}\right)\right)+\\ \sum_{i:z_{i}>0}(\log\Delta \left(\theta^{-1}\right)+\log\Delta \left(1+z_{i}\right)-\log\Delta \left(\theta^{-1}+z_{i}\right)) \end{array}$$

where *N* = total number of observations.

ZINB model is not nested within the NB model. Vuong statistic (VS) is utilized to differentiate between the NB and ZINB models. The VS can be determined as:

(8) $$\mathrm{VS}=\frac{\gamma \sqrt{N}}{S_{d}}$$

where

γ = mean of *Q* = $\ln\frac{M_{1}(.)}{M_{2}(.)}$

$M_{1}(.)$ = density function of the ZINB distribution

$M_{2}(.)$ = density function of the parent-NB distribution

$S_{d}$ = standard deviation, and

N = sample size

The goodness-of-fit of the ZINB model compared with other models is evaluated by using Akaike information criteria (AIC) and Bayesian information criteria (BIC) measures.

## Discussion of Model Results

This study develops a ZINB model to estimate the SES demand in Kelowna. Among the 65,534 observations, 55,704 observations—which is approximately 85% of the data—were randomly selected for developing the model and the rest were used for model validation. A summary statistic of the variables retained in the final ZINB model is reported in Table 1.

**Table 1.** Descriptive Summary of Independent Variables

Variable name	Definition	% or mean	SD
Temporal characteristics			
Summer	Dummy; If SES counts are made in summer = 1, else = 0	46.31	na
Fall	Dummy; If SES counts are made in fall = 1, else = 0	53.68	na
Weekend	Dummy; If SES counts are made on weekend = 1, else = 0	29.45	na
Weekday	Dummy; If SES counts are made on weekday = 1, else = 0	70.54	na
Mid-day	Dummy; If SES counts are made at mid-day (10:00 a.m.–3:00 p.m.) = 1, else = 0	21.21	na
Afternoon	Dummy; If SES counts are made in the afternoon (3:00–7:00 p.m.) = 1, else = 0	21.23	na
Evening	Dummy; If SES counts are made in the evening (7:00–10:00 p.m.) = 1, else = 0	20.89	na
Weather characteristics			
Rain	Dummy; If rainfall occurred = 1, else = 0	14.87	na
Wind speed	Wind speed in km/h	12.27	16.59
Relative humidity	Relative humidity in %	58.76	23.81
Transportation infrastructure characteristics			
Density of cycle track	Ratio of length of cycle track (km) to DA area (km^2^)	0.14	0.49
Ratio of length of bike lane to traffic lane	Ratio of length of bike lane to road in DA	0.37	0.30
E-scooter index (EI)	E-scooter index in DA	0.25	0.08
Number of bus stops	Number of bus stops in DA	3.62	4.32
Land use attributes			
Percentage of residential land use	Percentage of residential land use in DA	47.38	26.99
Land use index (LUI)	Land use index in DA	0.51	0.29
Higher percentage of residential land use	Dummy; If residential land use is greater than 50% in DA = 1, else = 0	26.99	na
Percentage of park and aquatic land use	Percentage of park and aquatic land use in DA	15.77	24.31
Urban centers	Area of urban centers (km^2^) in DA	0.05	0.13
Mean elevation	Mean percentage rise in elevation in DA	3.28	3.76
Neighborhood features			
Percentage of high-rise apartments	Percentage of apartments greater than five stories in DA	2.14	8.20
Average age	Average age of population in DA	43.46	6.87
Percentage of employed	Percentage of population in DA who are employed	92.98	3.62
Density of hotels	Ratio of number of hotels to DA area	1.42	4.97
Density of recreational activity points	Ratio of number of recreational activity points to DA area	2.78	9.61

*Note*: DA = dissemination area; SD = standard deviation; SES = shared e-scooter services, na = not applicable.

### Goodness-of-Fit Measures

For comparison purposes, in addition to the ZINB model, this study estimates the following models: Poisson, NB, hurdle Poisson (HP), hurdle negative binomial (HNB), and zero-inflated Poisson (ZIP) models. In the case of the goodness-of-fit measures, the ZINB model is compared with the NB model using the VS measure. A value greater than 1.96 for the VS favors the ZINB model. Results suggest that VS value for testing the ZINB versus NB model is 14.8 (Table 2). Therefore, the ZINB model outperforms the NB model. Further comparative results suggest that the ZINB model shows a lower BIC value compared with the other models. For example, BIC values for the ZINB, ZIP, HNB, HP, and Poisson are 10,756.1, 23,480.4, 11,162.7, 24,221.7, and 27,781.5, respectively. Similarly, the ZINB model shows a lower AIC value than the other models. Since the model with lower BIC and AIC measures fits the data best, the ZINB model is selected as the final model for further discussion. The estimation results from the ZINB model are presented in Table 3. It is found that the majority of the parameters show a statistical significance at least at the 1% significance level.

**Table 2.** Comparison of Goodness-of-Fit Measures of the Demand Models

Model	Log-likelihood function	Akaike information criteria (AIC)	Bayesian information criteria (BIC)	Vuong statistic (VS)
ZINB	−5,311.6	10,679.2	10,756.1	14.8
ZIP	−11,676.1	23,406.3	23,480.4	10.4
HNB	−5,514.9	11,086.0	11,162.7	na
HP	−12,046.8	24,147.6	24,221.7	na
NB	−6,165.9	12,363.8	12,407.7	na
Poisson	−13,855.1	27,740.3	27,781.5	na

*Note*: ZINB = zero-inflated negative binomial; ZIP = zero-inflated Poisson; HNB = hurdle negative binomial; HP = hurdle Poisson; NB = negative binomial; na = not applicable.

**Table 3.** Parameter Estimation Results of the Zero-Inflated Negative Binomial (ZINB) Model

Attributes	Coefficient	*t*-Stat
Results of the count component		
Constant	−7.84	−3.86^***^
Dispersion parameter	1.24	23.46^***^
Temporal and weather characteristics		
Summer	0.88	10.99^***^
Mid-day × weekend	0.44	3.04^***^
Afternoon × weekday	0.36	3.33^**^
Rain	−0.26	−2.59^***^
Wind speed × fall	−0.006	−1.57
Transportation infrastructure attributes		
Density of cycle track	0.78	5.15^***^
E-scooter index (EI)	3.51	3.92^***^
Ratio of length of bike lane to traffic lane	0.34	2.53^**^
Land use attributes		
Land use index (LUI)	0.41	1.69^*^
Urban centers	6.27	23.39^***^
Mean elevation	−0.35	−18.69^***^
Neighborhood features		
Density of hotels × weekend	0.01	3.14^***^
Percentage of employed	0.12	5.88^***^
Average age	−0.15	−16.74^***^
Results of the zero-inflation component		
Constant	−1.16	−2.24^**^
Temporal and weather characteristics		
Mid-day × weekend	−0.59	−4.28^***^
Evening × weekday	−0.51	−4.19^***^
Relative humidity × summer	0.009	5.01^***^
Wind speed × fall	0.01	3.40^***^
Transportation infrastructure and land use attributes		
E-scooter index (EI)	−2.78	−3.49^***^
Percentage of residential land use	0.03	21.64^***^
Number of bus stops × higher percentage of residential land use	−0.19	−5.41^***^
Percentage of park and aquatic land use	−0.03	−23.07^***^
Neighborhood features		
Density of recreational activity points	−0.04	−19.19^***^
Percentage of high-rise apartments	−0.06	−9.11^***^
Average age	0.10	8.77^***^

*** significance at 1% level

** significance at 5% level

* significance at 10% level.

### Parameter Estimation Results of the ZINB Model

#### Results of Count Component of the ZINB Model

The count component of the ZINB model confirms the effect of temporal, weather, transportation infrastructure, land use, and neighborhood characteristics on SES demand (Table 3). Model results reveal that the dispersion parameter is statistically significant. In the case of temporal characteristics, variable representing the summer season shows a positive sign. This indicates that the demand for SES is more likely to increase during the summer. Similarly, results suggest that there is a higher probability of increased demand for SES during the mid-day of weekends and afternoon of weekdays. This indicates that individuals might use SES for recreational purposes during the mid-day of weekends and for commuting purposes during the afternoon peak hour of weekdays. In the case of weather characteristics, rainfall is found to have a negative impact on SES volume. This implies that demand might be lower when rainfall occurs. A similar negative relationship is observed for wind speed coupled with fall season, suggesting that riding e-scooters in windy weather conditions during the fall season might not be attractive.

Results further reveal that transportation infrastructure attributes are found to have substantial influence on SES demand. For example, the variable representing the density of cycle track shows a positive relationship. This implies that SES demand might remarkably increase in the presence of cycle tracks. Cycle tracks are dedicated and protected bike infrastructure available for e-scooters, which might attract users to ride SES. This is because individuals riding e-scooters on cycle tracks are less vulnerable to collisions and feel safer. The variable representing the EI also positively influences demand. This is anticipated because EI signifies the overall e-scooter friendliness of an area. As a result, higher EI is more likely to increase demand. Similarly, the variable representing the ratio of length of bike lane to traffic lane shows a positive sign.

Among the land use attributes, the LUI shows a positive sign, implying that users might be attracted to ride SES in areas of heterogeneous land use mix. This is expected, as heterogeneous land-use areas allow individuals to participate in diverse activities. The variable representing the urban centers also exhibits a positive relationship. Urban centers are the hub of government, business, and personal services. Therefore, SES demand might increase within the urban centers. Conversely, a negative relationship is observed in the case of the variable representing the mean elevation. This reveals that areas with higher mean elevation might observe lower SES demand. This may be explained by steeper grades being more difficult for e-scooters to negotiate. Riding in areas with higher elevation also puts extra pressure on the battery and motor, eventually reducing the performance and efficiency of e-scooters.

In the case of neighborhood features, density of hotels interacted with weekend shows a positive sign. This implies that there is a higher likelihood of increased demand for SES on weekends in neighborhoods with a higher density of hotels. This can be explained by tourist users staying at hotels who might consider using SES for recreational purposes during the weekends. Similarly, a positive relationship is revealed for the variable representing the percentage of employed people in the neighborhood. This indicates that neighborhoods with higher percentage of employed people are more likely to induce higher demand for SES. Interestingly, the variable representing the average age exhibits a negative relationship. This suggests that neighborhoods with younger population are more likely to show higher SES demand.

#### Results of the Zero-Inflation Component of the ZINB Model

The zero-inflation component of the ZINB model also confirms the effects of temporal, weather, transportation infrastructure, land use, and neighborhood characteristics on SES demand (Table 3). The results of the zero component are consistent with the findings from the count component of the model. For example, among the temporal and weather characteristics, mid-day on weekends is negatively associated with the probability of zero SES demand. This reveals that there is higher propensity of e-scooter volume during the mid-day on weekends. This is expected as individuals are more likely to use SES for recreational purposes during the weekend. Similarly, the variable representing evening interacted with weekday exhibits a negative sign. This implies that demand is less likely to be zero during the evening of weekday. Individuals might use SES for returning home from their workplace in the evening on weekdays. In the case of weather attributes, the variable representing relative humidity in summer is positively associated with the propensity of zero demand. This is expected, as higher humidity in the summer makes it feel hotter than the actual air temperature, which is not the ideal weather condition for riding e-scooters. A similar positive relationship is observed for wind speed during the fall, implying that higher wind speed in the fall season might increase the likelihood of zero demand.

In the case of transportation infrastructure and land use attributes, EI shows a negative relationship. This indicates that there is a higher probability of SES demand in the areas with a higher EI. However, the percentage of residential area exhibits a positive sign, implying that higher proportion of residential land use increases the probability of being in zero-demand state. Interestingly, number of bus stops coupled with a higher percentage of residential area influences the zero-demand state negatively. This indicates that SES might solve the first- and last-mile problem of urban trips by connecting the users with increased accessibility to public transit in residential areas. Furthermore, the variable representing the percentage of park and aquatic land use reveals a negative relationship. It suggests that areas with parks and lakes might be attractive for SES users.

Among the neighborhood characteristics, density of recreational activity points is negatively associated with the probability of zero demand. This reveals that neighborhoods with higher density of recreational activity points are more likely to induce demand. A similar positive relationship is observed for the variable representing the percentage of high-rise apartments in the neighborhood. Interestingly, the variable representing the average age in the neighborhood shows a positive sign. This suggests that an older population in the neighborhood is less likely to induce demand. Younger individuals might find SES more attractive.

### Model Validation Results

**Figure 3.**
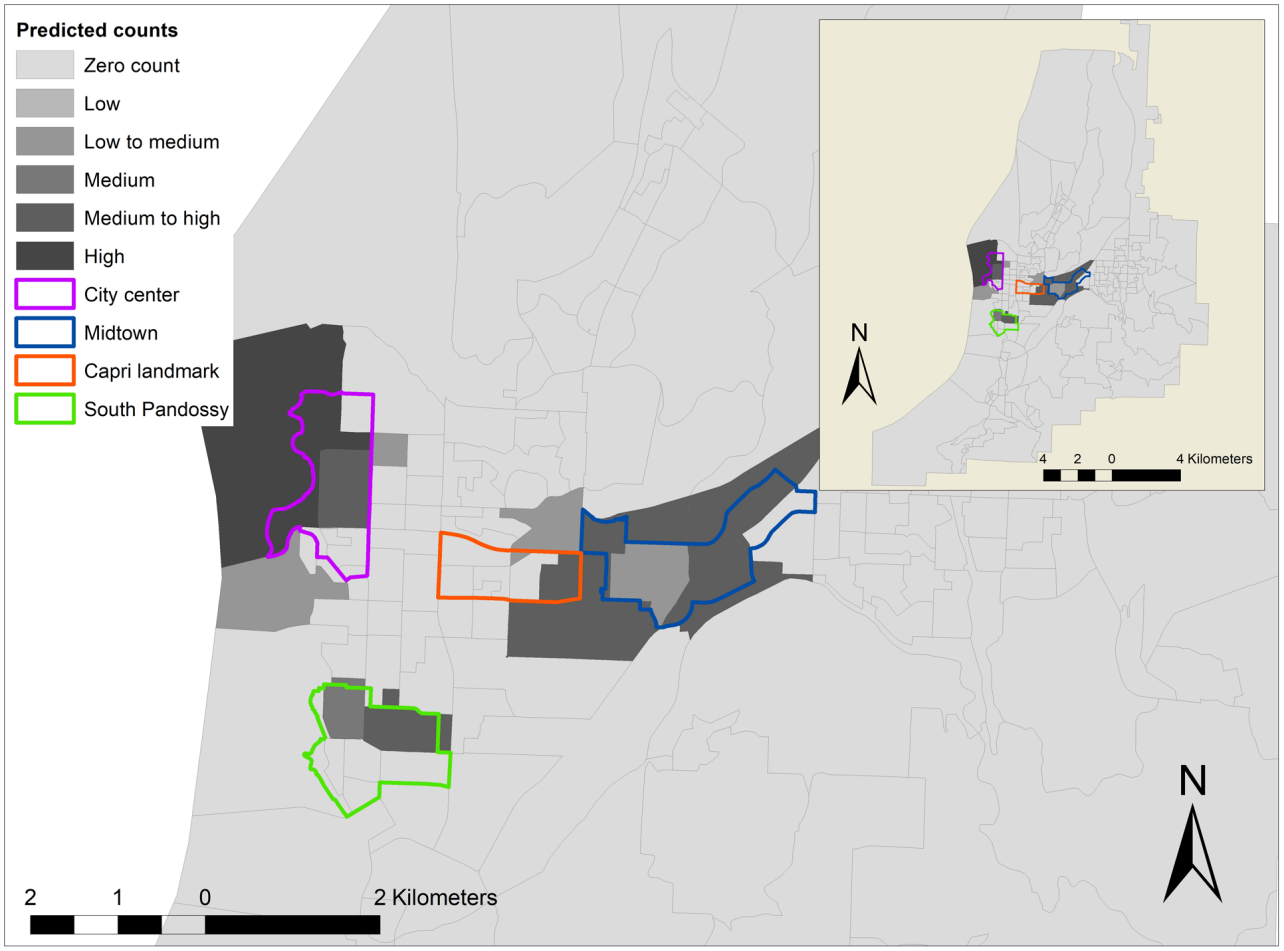
Predicted shared e-scooter service (SES) demand in Kelowna.

The ZINB model results are validated using a hold-out sample to evaluate the predictive performance of the model. The hold-out sample includes 9,830 observations. The validation results suggest that the value of adjusted pseudo R-squared is 0.53 for the ZINB model which is higher than the HNB (i.e., 0.52) and NB (i.e., 0.47) models. Therefore, ZINB model reveals a better fit of the model for the validation sample. The predictive performance of the ZINB model is also compared with the HNB and NB models (Table 4). Validation results indicate a higher prediction precision for the ZINB model than that of other models. For example, the mean absolute deviation (MAD) value for the count component of the ZINB model is 0.303, HNB model is 0.319, and NB model is 0.337. The smaller the value of MAD, the higher the accuracy of prediction. Furthermore, the absolute percent error (APE) for zero counts is smaller for the ZINB model (2.64%) than for the HNB model (2.85%) and NB model (3.44%). Therefore, it can be concluded that the ZINB model offers satisfactory results for predicting the demand for SES for the validation sample. Figure 3 illustrates the predicted demand for SES aggregated in the DAs of Kelowna. The figure reveals some aspects identical to the SES demand observed in Kelowna. For instance, the density of predicted demand is very high near the Okanagan Lake adjacent to the downtown area. Moreover, a secondary peak of demand is predicted within the urban city center. In the case of observed demand, a similar trend is noticed (Figure 2). This further confirms the satisfactory model fit and prediction accuracy of the ZINB model.

**Table 4.** Model Validation Results

	Predictive performance measures	
Model	Count of shared e-scooter services (SES)	Zero count
	Mean absolute deviation (MAD)	Absolute percent error (APE) (%)
ZINB	0.303	2.64
HNB	0.319	2.85
NB	0.337	3.44

*Note*: ZINB = zero-inflated negative binomial, HNB = hurdle negative binomial, NB = negative binomial.

$\mathrm{MAD}=\frac{\sum_{n=1}^{N}\left|Z_{\mathrm{pred}}-Z_{\mathrm{obs}}\right|}{N},\mathrm{APE}=\frac{\left|P_{0}-O_{0}\right|}{O_{0}}*100$ .

where $X_{\mathrm{pred}}$ $Z_{\mathrm{pred}}=$ predicted count; $Z_{\mathrm{obs}}$ = observed count; *N* = total observations.

$P_{0}$ = number of zero count predicted; $O_{0}$ = number of zero count observed.

## Conclusions

This study investigates the demand for SES; specifically, it examines the spatio-temporal variation of SES demand. A ZINB model is developed using the counts of SES trip origins at the DA level for Kelowna, Canada. One of the reasons for adopting the ZINB model is that it addresses the existence of excess zero counts in the data. The excess zeros are categorized as structural (i.e., DAs always with zero counts) and sampling zeros (i.e., DAs with zero counts only at specific time periods). The ZINB model is a two-component model, where the zero-inflated component follows a binary distribution to account for excess zeros. The count model component adopts a NB distribution and accommodates the over-dispersion characteristics of data resulting from excess zeros. One of the key aspects of this research is to investigate the effects of temporal attributes such as time of day, week, season, and weather characteristics, as well as spatial attributes such as transportation infrastructure, land use, and neighborhood features.

In addition to the ZINB model, NB, ZIP, HNB, HP, and Poisson models are also estimated for comparison purposes. The goodness-of-fit measures suggest that the ZINB model outperforms other methods on AIC, BIC, and VS measures. The model results of the count component of the ZINB model suggest that demand is more likely to be higher during the mid-day of weekends, afternoons of weekdays, and summer season. In contrast, rainfall and higher wind speed during the fall season might induce a lower demand. Among the transportation infrastructure attributes, density of cycle track, EI, and a higher ratio of bike lane to traffic lane are positively associated with demand. In the case of land use attributes, demand is likely to be higher in areas with higher LUI and in urban centers. However, demand might be lower in areas with a higher mean elevation. In the case of neighborhood features, demand is more likely to be higher in areas with a higher employment rate, and areas with a higher density of hotels—specifically during the weekends. In contrast, neighborhoods with a larger share of older adults are less likely to induce higher demand.

The results of the zero-inflated component of the model are consistent with the count model. For example, results suggest that demand is less likely to be zero during the mid-day of weekends and evenings of weekdays. Furthermore, higher EI, higher transit accessibility in the residential areas, higher percentage of park and aquatic areas, higher density of recreational activity points, and higher percentage of high-rise apartments might induce demand. On the other hand, higher relative humidity during summer, higher wind speed during fall, higher percentage of residential land use, and neighborhoods with older age population might induce zero volume of SES. Furthermore, the ZINB model is validated using a 15% hold-out sample. The validation indicators such as the MAD and APE measures suggest that the ZINB model shows a higher prediction precision. Model results are further validated by comparing the illustration of the predicted demand for SES with the observed demand. The validation results suggest that the performance of the model is reasonably satisfactory.

This study has certain limitations. For example, the demand model is developed based on a small sample of data from Kelowna, British Columbia. The majority of the data was utilized to build the model; only 15% of the data was used as hold-out sample for model validation. Future research should focus on collecting more data for longer periods of time so that the data can be utilized as hold-out sample for improved model validation. Furthermore, this research does not test the spatial transferability of the developed model to cities other than Kelowna. One of the approaches could be to apply the e-scooter demand model for another city and assess the performance by estimating the predictive accuracy of the transferred model for the application context. In such case, goodness-of-fit measures of the predictive accuracy such as root mean square error (RMSE) (*34*) and/or transfer index (TI) (*35*) for the application context can be used. If the results of the full spatial transferability of the model are not satisfactory, then some of the parameters of the model might need to be calibrated for the application context and the prediction accuracy can then be tested. One of the key considerations while testing the transferability is the data requirement for the application context. To test and confirm the transferability of the developed e-scooter demand model, data including the temporal and weather characteristics, transportation infrastructure and land use attributes, and neighborhood features need to be collected for the application context. In addition to collecting similar data, it is also important to make consistent assumptions while deriving and/or testing variables for the application context. For example, land use attributes need to be generated at the spatial resolution of the DA for the application context which is similar to that of the base model. In the case of statistically evaluating the transferability of the model to other application contexts, different statistical tests of model equivalence can be performed (*36*). For example, model equality test statistic can be estimated to examine the transferability of a subset of variables while allowing for several variables to be different (e.g., weather). Transferability test statistic can also be calculated by using the log-likelihood of the base and transferred demand models (*37*). Another limitation is that this study could not consider the availability of e-scooters in DAs because of the unavailability of such data. Future research should focus on collecting data related to e-scooter availability to incorporate this factor in the model. Furthermore, this study does not explicitly account for whether the demand for SES is induced (i.e., demand originated from available SES on the streets) or latent (i.e., demand originated from SES not being at the area where an individual wished to make a trip). Future research should focus on identifying the latent demand originating from the e-scooters not being at the SAs where the user desires to ride an SES. Overall, this study provides important insights into how SES demand varies spatially and temporally. The findings of this study will assist in developing plans and policies to effectively promote SES at different times of day, week, and season, as well as at different locations of the city.
